# Minimization of the threshold voltage parameter of the co-doped ZnO doped liquid crystals by machine learning algorithms

**DOI:** 10.1038/s41598-023-39923-8

**Published:** 2023-08-07

**Authors:** Gülnur Önsal, Onur Uğurlu, Ümit H. Kaynar, Deniz Türsel Eliiyi

**Affiliations:** 1 Department of Fundamental Sciences, Izmir Bakircay University, 35665 Izmir, Turkey; 2 Department of Industrial Engineering, Izmir Bakircay University, 35665 Izmir, Turkey

**Keywords:** Materials science, Electronic properties and materials, Nanoparticles, Computer science

## Abstract

This study aims to examine the influence of the co-doped semiconductor nanostructure (Al-Cu):ZnO on the electro-optical properties of the E7 coded pure nematic liquid crystal structures and minimize the threshold voltage of pure E7 liquid crystal. To determine the ideal concentration ratios of the materials for the minimum threshold voltage, we employed different machine learning algorithms. In this context, we first produced twelve composite structures through lab experimentation with different concentrations and created an experimental dataset for the machine learning algorithms. Next, the ideal concentration ratios were estimated using the AdaBoost algorithm, which has an $R^2$ of 96% on the experimental dataset. Finally, additional composite structures having the estimated concentration ratios were produced. The results show that, with the help of the employed machine learning algorithms, the threshold voltage of pure E7 liquid crystal was reduced by 19% via the (Al-Cu):ZnO doping.

## Introduction

Zinc Oxide (ZnO) is an exhaustively researched material as its bandgap of around 3.3 eV renders it attractive for optoelectronic applications such as Light-emitting diodes (LEDs) and solar cells. Its bandgap also lets it to absorb photons with high energies, making it suitable for photodetection and photocatalysis. Moreover, its non-toxic structure, chemical and thermal stability, high electron mobility, inexpensive production cost, and unique electrical-optical and dielectric characteristics at room temperature are this material’s additional advantages^1,2^. For this reason, ZnO became a popular material for short wavelength opto-electronic devises, transistors, photo diodes and Liquid Crystal (LC) based sensors and laser applications^3^. The ZnO structure can be doped with some elements such as Fe^4^, Cu^5,6^, Co^7^, Gd^8^, or Al^9^ to improve its optical and electrical properties. In the past few years, many studies have been conducted to examine the effect of co-dopants such as (Cu-Mg)^10^, (Cd-Ni)^11^, (Al-In)^12^, (Fe-Al)^13^, (Al-Cu)^14^ on the electro-optical properties of ZnO, and it was shown that the electro-optical properties of the ZnO nanomaterial are improved through co-doping.

Copper doped Zinc Oxide (Cu:ZnO) has lately attracted significant interest due to its unique optical and electrical properties. One of the main advantages of Cu:ZnO is its ability to enhance the optical properties of ZnO. The insertion of copper ions into the ZnO lattice leads to a shift in bandgap energy, which can result in a change in the optical absorption and emission properties^6,15^. This makes Cu:ZnO a promising material for optoelectronic applications such as ultraviolet (UV) LEDs and solar cells. Another advantage of Cu:ZnO is its ability to enhance the electrical properties of ZnO. The presence of copper ions in the ZnO lattice results in creation of additional electrons and hole carriers, which increases conductivity and mobility of the material. This makes Cu:ZnO attractive for electronic applications such as sensors and transistors. The doping of Aluminum into ZnO is a technique used to improve the electronic and optical properties of the material. Some advantages of this process include increased conductivity, improved optical absorption, and enhanced thermoelectric performance^16^. Potential applications of Aluminum-doped zinc oxides are very promising in the field of electronics, optoelectronics, thermoelectrics, biomedical and antimicrobial applications^17^. Due to these features, in this study Al and Cu were chosen as the elements to be doped to ZnO for the co-doped ZnO nanoparticle.

LCs have become increasingly popular in recent years due to their unique combination of liquid- and solid-like properties. One of the main reasons for the popularity of LCs is their ability to change their electro-optical properties in response to an applied electric field. This property is known as the electro-optic effect, and is widely used in Liquid Crystal Displays (LCDs), which are the most common application of LCs. In addition to LCDs, LCs have also been used in other electronic devices such as electro-optic modulators, sensors, and solar cells^18^. The doping of LCs can lead to a wide range of benefits, such as improved electro-optical properties, enhanced thermal stability and increased alignment properties. Dopants such as as metal oxides have been found to enhance the electro-optical characteristics of LCs^19^. Metal oxides used as dopants are typically transition metal oxides such as titanium dioxide ($TiO_{2}$)^20^, zinc oxide (*ZnO*)^21^, and barium titanate ($BaTiO_{3}$)^22^. The doping ZnO nanoparticles (NPs) into LC change the molecular orientation and decrease the threshold voltage ($V_{th}$), which leads lower power consumption^23,24^. Particularly, the doping with low concentrations of ZnO enhanced the dielectric and electro-optical responsiveness.

The process of material creation is time-consuming, exhaustive and expensive. Numerous samples are required to create a composite material with the desired properties, resulting in increased material costs. Thus, in this study, we adopt a quasi-experimental methodology, which combines physical experiments with strong machine learning-based prediction algorithms. Designing robust prediction models can provide helpful insights into the properties of samples that have not been experimentally produced.

The usage of machine learning (ML) techniques in materials science has increased thanks to their capability to handle non-linear relationships^25–27^. Especially in recent years, several researchers have used ML algorithms to predict certain properties of LCs, such as average order parameter, sample temperature, cholesteric pitch length, and phase transition temperature^28–30^. In addition to these works, some researchers used ML algorithms to estimate the threshold voltage of some materials. Moparthi et al. employed a ML approach for assessing the process variability impact on the threshold voltage of silicon-on-insulator junctionless transistor^31^. Mishra et al. presents a genetic algorithm-based deep learning algorithm to estimate the threshold voltage of gallium nitride-based high electron mobility transistor^32^. They used maximum transconductance on current and subthreshold slope as input parameters, and reported an $R^2$ of 0.978.

**Figure 1:**
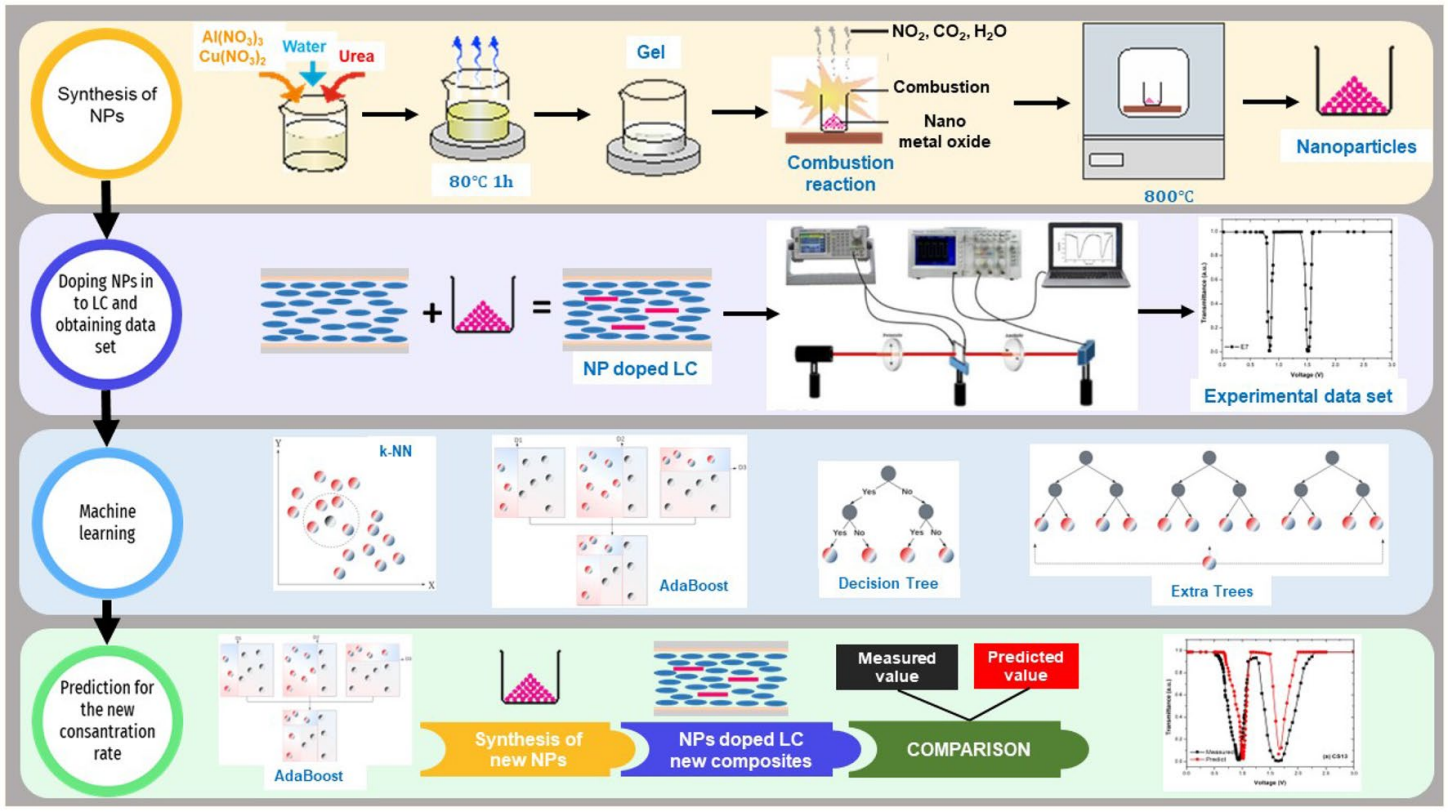
General overview of this study.

The main aim of this study is to minimize the threshold voltage of the E7-coded pure nematic liquid crystal by doping it with co-doped semiconductor nanostructure (Al-Cu):ZnO. However, determining the optimal concentration ratios of the materials used to form the composite structure with a low $V-{th}$ is a fundamental problem. To handle this challenge, initally, (Al-Cu):ZnO semiconductor nanomaterials with different concentrations were synthesized and the synthesized nanomaterials were added to E7 nematic liquid crystal at 1%, 3% and 5% weight ratios. In this way, we obtained 12 composite structures and created an experimental dataset by utilizing an electro-optical transmittance system on these composites. Next, we developed a reliable prediction model using ML algorithms to estimate the threshold voltage of the composites. After training our prediction model on the experimental dataset, we tried to find the optimal concentration ratios of the materials which would yield the minimum threshold voltage. Then, three new composite structures were obtained using the determined concentration ratios by the prediction models, and the electro-optical properties of these composite structures were investigated. Figure 1 represents the general methodology followed in this study.

The main contribution of this work is the established material design methodology to produce composites with desired properties. Commonly, LC has been dispersed with pure ZnO^33,34^ or single-doped ZnO nanomaterial^35^. In the literature, co-doped ZnO and LC were examined in only one study. Eskalen et al. investigated the effect of (Multi-Walled Carbon Nanotube-MWCNT; Silicon Dioxide-SiO$_{2}$) MWCNT/SiO$_{2}$@ZnO nanocomposite on the thermal and electrical behavior of the E7 nematic liquid crystal^21^. The authors only examined the predetermined concentration ratios of nanocomposites and reported the electrical behavior of the E7 nematic liquid crystal. Thus, the main novelty of our study is the usage of machine learning algorithms to determine the ideal concentration ratios of nanocomposites with the lowest voltage values.

## Materials and methods

### Synthesis of co-doped ZnO nanoparticles

Al-Cu doped ZnO materials were prepared by the gel-ignition method. Zinc nitrate $(Zn(NO_{3})_{2}$, >99%, Merck) and urea ($NH_{2}CONH_{2}$) as fuel were used for ZnO synthesis. High purity Aluminum Nitrate ($Al(NO_{3})_{3}$, >99%, Merck) and Copper(II) Nitrate ($Cu(NO_{3})_{2}$, >99%, Merck) were used for the co-doped samples. All items were weighed using stoichiometric proportions. In a quartz beaker, 10 ml of distillated water was used to dissolve nitrates. Urea was put in the beaker and the mixture was stirred for 1 hour at 80 $^{\circ }$C using a magnetic stirrer. The top of the beaker was then removed and the mixture was stirred to evaporate excess water at the same temperature until a gel-like consistency was attained^36^. Following the evaporation of the water, a flame combustion reaction concluded the synthesis process. The product was heated to 800 $^{\circ }$C in a muffle furnace in order to eliminate organic impurities in the form of fly ash, and change unstable crystalline phases into stable phases^37^. The obtained materials were stored in a desiccator to prevent interaction with atmospheric gases. Thus, four NP containing different ratios of Al and Cu were synthesized. The list of these NPs is provided in Table 1.

**Table 1:** Synthesized nanoparticles.

Nanoparticle	Host crystal	Ion	Ion	Fuel
NP1	ZnO	1%Al	1%Cu	Urea
NP2	ZnO	1%Al	8%Cu	Urea
NP3	ZnO	8%Al	1%Cu	Urea
NP4	ZnO	8%Al	8%Cu	Urea

### Preparations of the nanoparticle doped LC composites

**Figure 2:**
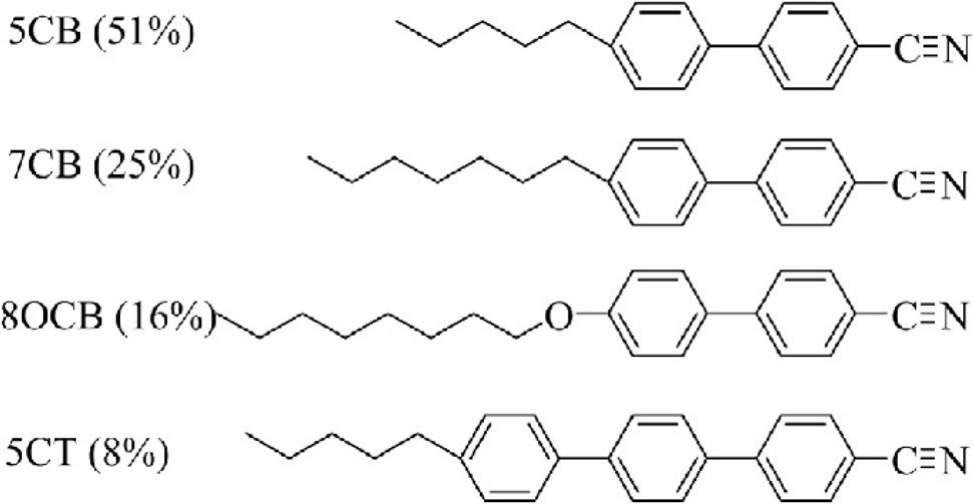
The molecular structures of E7 nematic liquid crystal.

A room temperature nematic LC E7 from Merck, compositions: 4-cyano-4$'$-n-pentyl-biphenyl (51%), 4-cyano-4$'$-n-heptyl-biphenyl (25%), 4-cyano-4$'$-n-oxyoctyl-biphenyl (16%) and 4-cyano-4$''$-n-pentyl-biphenyl (8%) with dielectric anisotropy (${\Delta \varepsilon '}$) = $+$13.8, birefringence (${\Delta }$n)= 0.20 and nematic-isotropic temperature ($T_{N-I})= 60.5^{\circ }$C were used for the experiments. The molecular structures of E7 nematic liquid crystal are depicted in Fig. 2.

Four synthesized NPs were doped in E7 in 1%, 3% and 5% wt/wt concentration, and 12 different samples were obtained, which are listed in Table 2. Chloroform was also added to these samples as a solvent to dissolve the NPs. The samples were maintained in an ultrasonic bath at 50 $^{\circ }$C for 6 hours to ensure a homogeneous dispersion. The samples were then heated to 60 $^{\circ }$C for a period to remove the solvent (chloroform), before they were cooled to room temperature for 24 hours. LC cells with planer alignment were filled with samples using the capillary technique at a temperature around 5 $^{\circ }$C higher than the isotropic temperature. The Indium Tin Oxide (ITO)-coated 7.7 $\mu$m thick LC cells (produced by Instec, USA) have a sheet resistance of 100 ${\Omega }$.

**Table 2:** The composite structures.

Composite structure	Concentration ratios
CS1	E7+1%NP1
CS2	E7+1%NP2
CS3	E7+1%NP3
CS4	E7+1%NP4
CS5	E7+3%NP1
CS6	E7+3%NP2
CS7	E7+3%NP3
CS8	E7+3%NP4
CS9	E7+5%NP1
CS10	E7+5%NP2
CS11	E7+5%NP3
CS12	E7+5%NP4

Using an electro-optical switching technique, electro-optical measurements were performed at 1 kHz square wave frequency and 0–20 $V_{pp}$ voltage range. In this technique, a He-Ne laser beam with a wavelength of 632.8 nm was used as the input signal, and the beam passed through the LC cell positioned between a polarizer and an analyzer that were in a crossed position. During this process, the voltage value was increased by 10 mV increments with a function generator, and the detected light intensity signal was recorded.

### Machine learning algorithms

Machine learning algorithms are methods that perform a learning process to create a model for describing the relationship between particular input and output data. The learning process is accomplished by adjusting the hyper-parameters of the model to minimize prediction error on an independent validation dataset. Two different approaches can be applied in ML algorithms, namely classification and regression. In classification, the sample is labeled with one of the predetermined class labels, whereas a numeric value is estimated as output in regression. As we aim to predict the transmittance values in this study, we used the regression version of ML algorithms.

Four different ML regression algorithms (k-Nearest Neighbor, Decision Tree, Extra Tree, and AdaBoost) were employed on the experimental dataset for estimating the threshold voltage. To run the ML algorithms, Scikit-learn, which is a widely used Python machine learning library, was used. The prediction performances of the algorithms were evaluated using *k*-fold cross-validation. The hyper-parameters of algorithms are one of the most important factors in their predictive performance. Hence, a grid search technique was carried out within Scikit-learn to determine the optimal levels of hyper-parameters for the algorithms.

### Employed algorithms

k-Nearest Neighbor (kNN) is a straightforward ML algorithm, which works on the assumption that similar data points are close to each other in terms of distance^38^. To estimate the value of a new data point, the distance of that point to the existing ones are calculated, and its *k* close neighbors are checked. The most critical hyper-parameters that affect the performance of the kNN algorithm are the number of neighborhoods and the distance metric. In this study, the number of neighbors was optimized in [1, 2, 3,..., 20] and the distance metric was optimized in [minkowski, euclidean, manhattan] metrics to design the kNN algorithm.

The decision tree (DT) algorithm uses a tree structure to represent a set of possible decision paths and an outcome for each path. The nodes in the tree represent an event or choice, and the branches represent the decision rules or conditions^39^. The first node of the decision tree is called the root node, and the lowest nodes are called the leaf nodes. The nodes between the root and the leaves are called the interval nodes. The leaf nodes provide the final prediction. For regression problems, the DT algorithm uses decision trees to predict the numeric outcomes by repeatedly dividing the tree. The most critical hyper-parameter of the decision tree algorithm are the maximum depth of the tree. For designing the DT algorithm in this study, the maximum depth of the tree was optimized in [2,3, 4, 5, ..., 20].

Extra trees (EXT) is an ensemble learning algorithm. Ensemble learning is one of the new trends in ML research, which uses the same learning model with different training sets or combines more than one learning model instead of using a single model on the same training set. Ensemble learning approaches usually outperform traditional learning algorithms^40^. EXT uses a random subset of features to train each base learner by using the whole training set for training each tree^41^. Besides, random branching is preferred in EXT instead of calculating the locally optimal separation using decision criteria. The most critical hyper-parameters of the EXT algorithm are the maximum depth of the tree and the number of estimators. For designing the EXT used in our study, the maximum depth of the tree was optimized in [2, 3, 4, ..., 20] and the number of estimators was optimized in [10, 20, 30, ..., 200].

**Figure 3:**
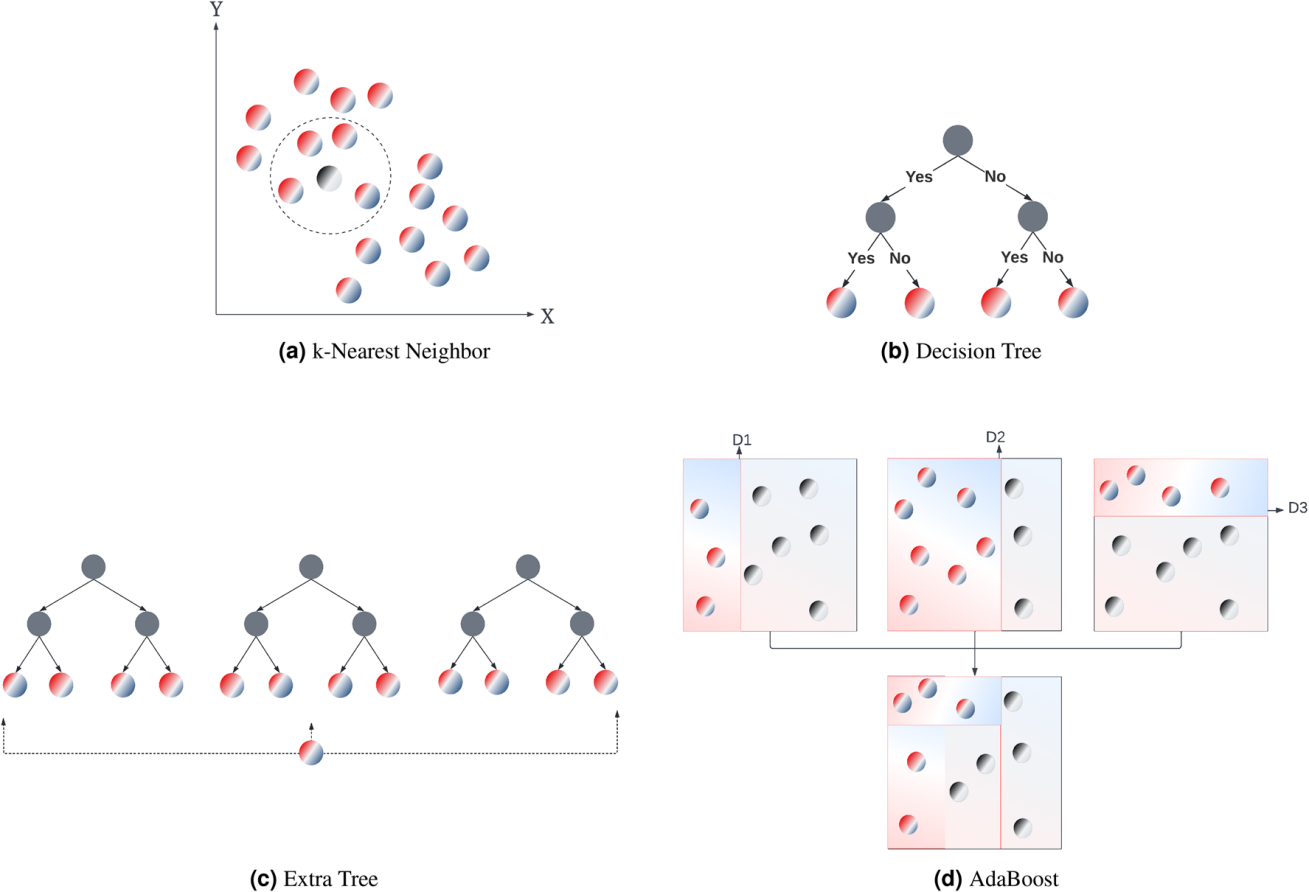
Illustration of the employed machine learning algorithms: (**a**) kNN, (**b**) DT, (**c**) ET and (**d**) AB.

AdaBoost (Adaptive boosting- AB) is another ensemble learning algorithm that is based on the boosting method. In the boosting method, the models are trained sequentially. Each model sees the previous model and learns from it. AB algorithm executes a large number of learning algorithms called weak learners, one after the other, to increase the prediction performance. The final estimate of the algorithm is obtained by the weighted average of the weak learners’ outputs^42^. At the beginning of the AB algorithm, the training samples start with equal weights. Then, at each iteration, the sample weights are changed separately and the learning algorithm is applied again to the re-weighted data. While the weights of the training samples incorrectly predicted by the model in the previous step are increased, the weights of the correctly-predicted samples are decreased. As the algorithm works, the weights of the hard-to-guess samples gradually increase. Thus, each subsequent weak learner is forced to focus on more complex cases. The most important hyper-parameters for AB are the base learner and the number of estimators. For designing the AB in this study, the number of estimators was optimized in [10, 20, 30, ..., 200], and the base learner was optimized in [kNN, DT, EXT]. Figure 3 illustrates the ML algorithms used in this study, and Table  3 lists the error metrics used for evaluating the performance of the algorithms (where *n* is the number of observations).

**Table 3:** Error metrics used for evaluating the ML algorithms.

Metric	Description	Equation
MAE	Mean absolute error	$\frac{1}{n}\sum _{i=1}^{n}|y_{actual}^i - y_{predict}^i|$
RMSE	Root mean square error	$\sqrt{\frac{1}{n}\sum _{i=1}^{n}(y_{actual}^i - y_{predict}^i)^2}$
$R^2$	Coefficient of determination	$\frac{\sum _{i=1}^{n}(y_{predict}^i - y_{mean})^2}{\sum _{i=1}^{n}(y_{actual}^i - y_{mean})^2}$

## Results

### Chemical characteristics of the nanoparticles

**Figure 4:**
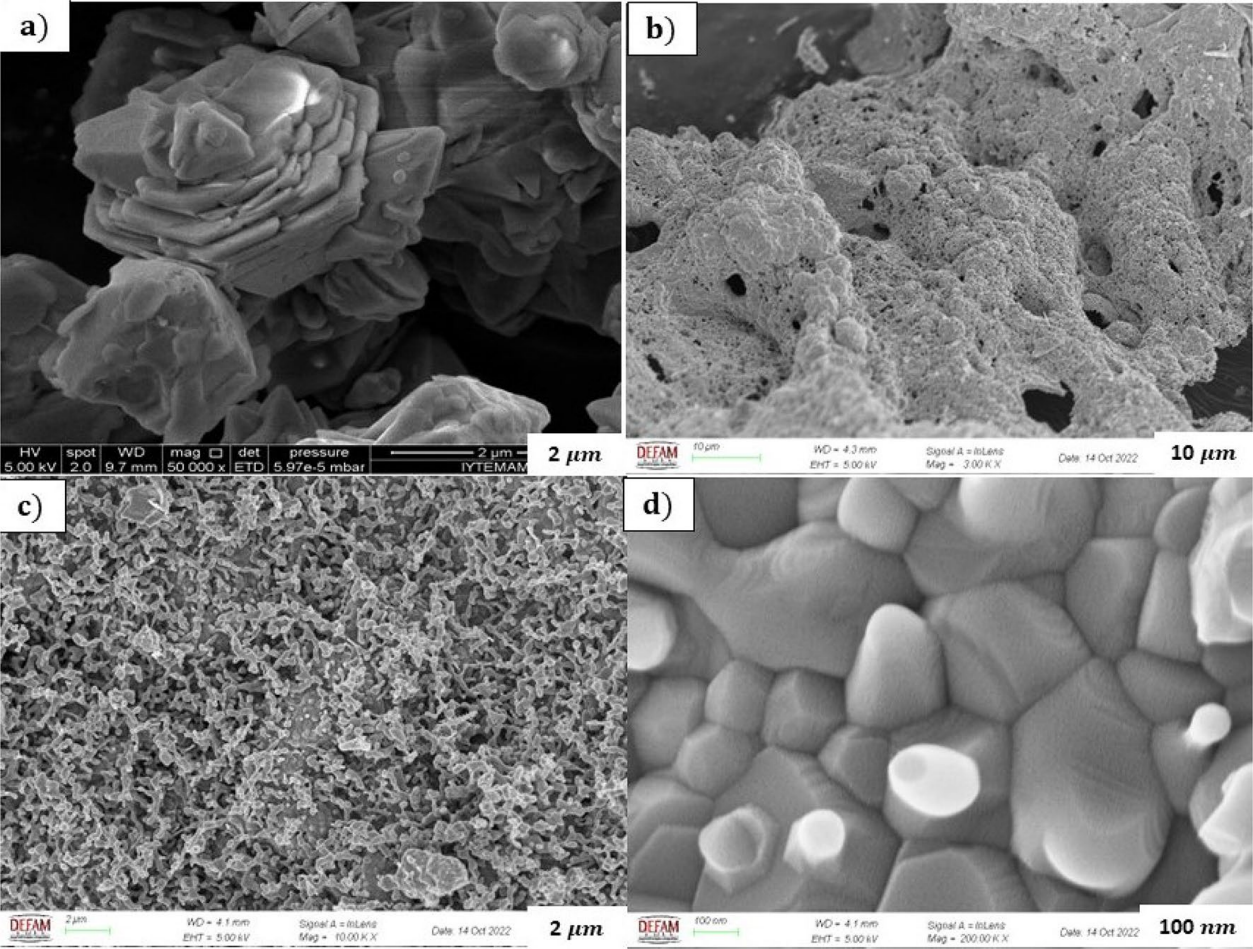
SEM images of the synthesized (**a**) pure ZnO and (Al-Cu):ZnO under different magnification (**b**) 10 $\mu$m (**c**) 2 $\mu$m (**d**) 100 nm.

Scanning electron microscope (SEM) images were analyzed to examine the morphological structure and characteristics of nanostructures synthesized with and without additives. The well-known hexagonal crystal structure of the undoped ZnO appears morphologically as plates in Fig. 4a^43^. Figure 4b–d show ZnO NPs doped with Al-Cu ions under different magnification. It is shown that the influence of the ignition reaction of the doping process in the doped structures boosts the ignition feature of the fuel. It is seen in Fig. 4b–d that the heat generated during the reaction increases the pore density and the gas output of the synthesized samples. Furthermore, these factors in combustion show that the hexagonal form in the sintered structure is deteriorated. According to the morphological structure, it is seen that the doping process changes many properties of the material, such as the reduction of the surface area and particle size^44^.

**Figure 5:**
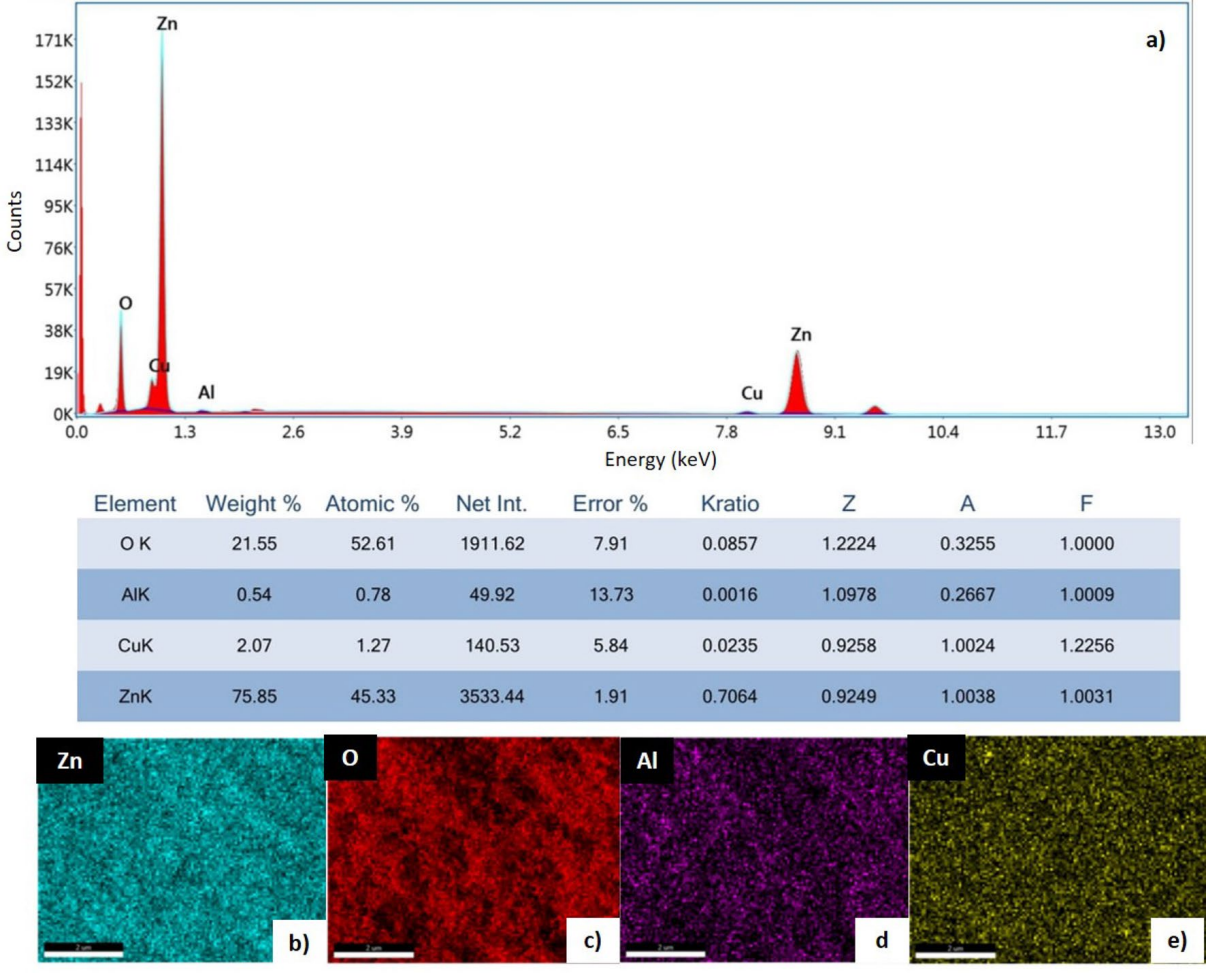
(**a**) EDX spectrum of the co-doped ZnO and elemental mapping of the (**b**) Zinc (Zn) (**c**) Oxygen (O) (**d**) Aluminum (Al) and (**e**) Copper (Cu).

According to the SEM-EDX/Elemental Mapping images of the synthesized ZnO nanoparticles, the distribution of Al and Cu additives in the structure are shown in Fig. 5. The mapping images confirm that the Al and Cu additives are homogeneously distributed in the main structure^45^.

**Figure 6:**
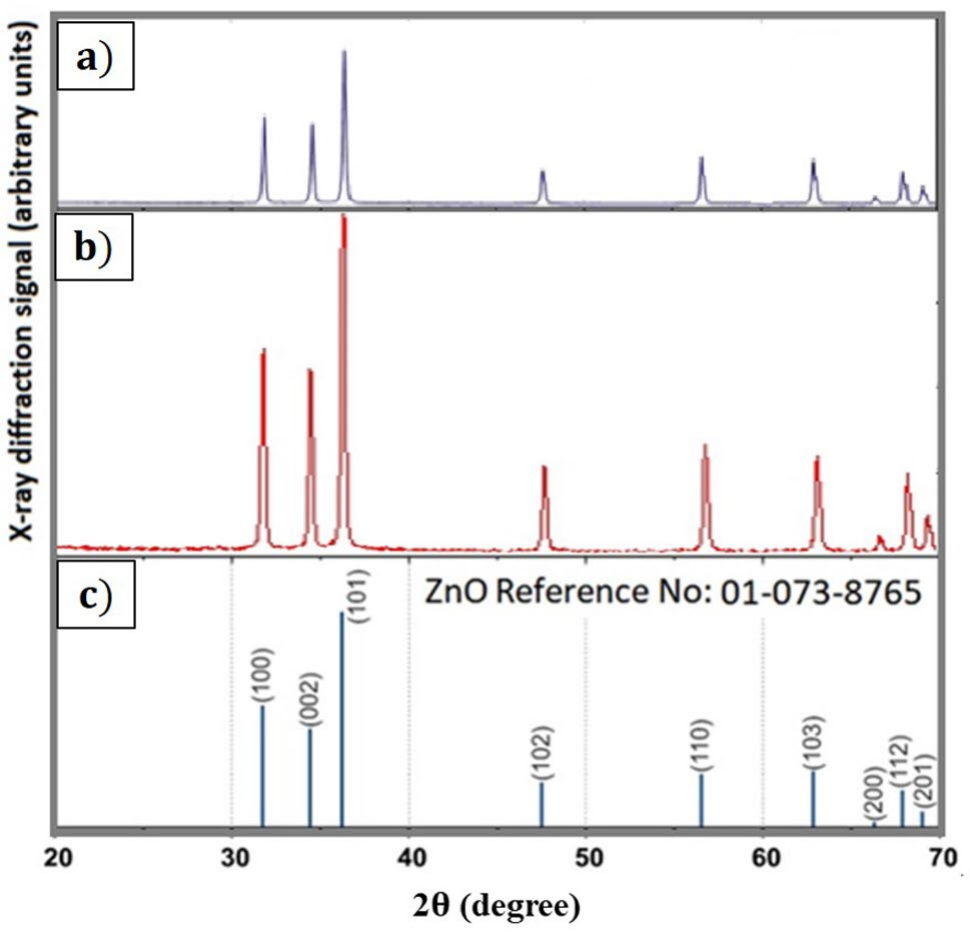
X-ray diffraction pattern for the synthesized (**a**) pure ZnO (**b**) co-doped ZnO and (**c**) ZnO reference No: 01-073-8765.

The crystal phase evaluation for the structure accuracy of all undoped and doped ZnO samples synthesized by the ignition reaction was determined by the X-ray diffraction (XRD) technique. Figure 6 depicts the reflection lines of the synthesized NPs, the reference reflection lines of the ZnO structure, and the Miller index reflection lines. Here, the crystal phase of the samples synthesized by the Ignition method corresponds to (a) the pure ZnO crystal and (b) the ZnO nanomaterial doped with Al and Cu. Although the miller does not disrupt a crystal structure according to the reflection lines, they cause stresses in the crystal lattice in the main structure of the doped materials. Nevertheless, no difference was observed in the structure^44^.

These results show us that the changes in particle size and charge balance in the doped synthesized structure cause electrostatic interaction of charged particles and change in surface potential.

### Detection of threshold voltage of the composites

Utilizing an electro-optical transmittance system, the electro-optical performance of the LCs was assessed. In this system, transmittance values corresponding to various voltage values were detected and transmittance-voltage plots were created. During the transmission measurement, the LC cells were positioned between crossed polarizers at a 45 $^{\circ }$ angle with the incident light’s optic axis. The intensity of light transmitted through a cell is given by the following equation^46^:

1 $$\begin{aligned} {T=\frac{1}{2}\left[ cos^2(\varphi _{1}-\varphi _{2})-sin2\varphi _{1}sin2\varphi _{2} sin^2\frac{\delta }{2}\right] }, \end{aligned}$$

where $\varphi _{1}$ and $\varphi _{2}$ are the angles between the orientation direction and the two polarizers, and $\delta$ is the phase retardation. The threshold voltage is defined as the voltage when the initial transmission of the cell begins to change, and is expressed with the following equation^19^:

2 $$\begin{aligned} {V_{th}=\pi \sqrt{\frac{K_{11}}{\varepsilon _0 \Delta \varepsilon '}} }, \end{aligned}$$

where $\Delta \varepsilon '$ and $K_{11}$ are defined as dielectric anisotropy and splay elastic constant parameters, respectively. $\varepsilon _0$ dielectric constant of the free space charge ($\varepsilon _0=8.85\times 10^{-14} F cm^{-1}$).

**Figure 7:**
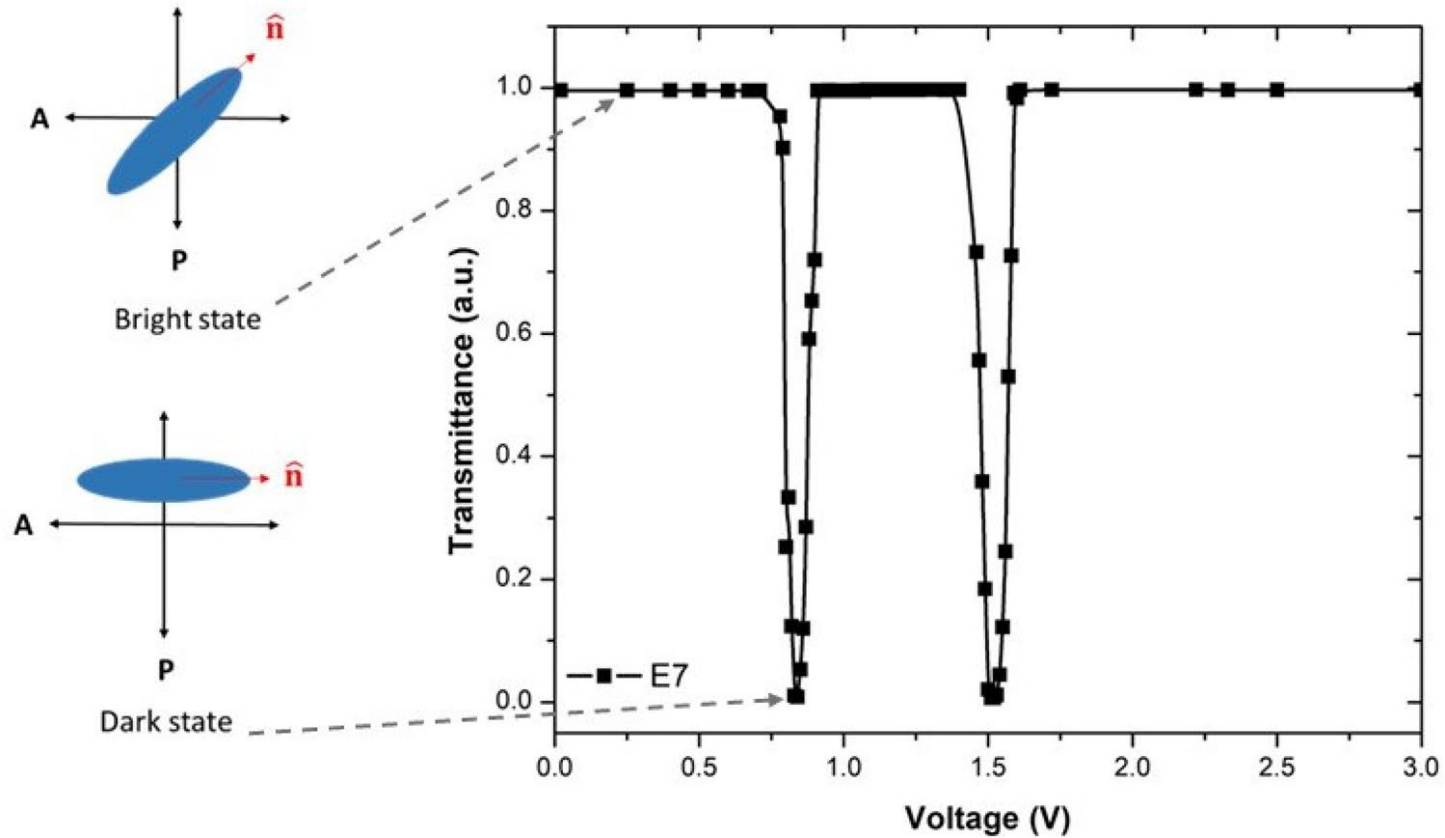
Transmittance-applied voltage plots of E7 nematic liquid crystal.

$V_{th}$ which is a crucial parameter for the LC is the minimal voltage value necessary to reorient LC molecules, and can be calculated from the Transmittance-Voltage (T-V) plots by determining the voltage value at which the transmitted light intensity changes by 10%. Normalized T-V plot for E7 pure liquid crystal is given in Fig. 7. Using the Fig. 7, $V_{th}$ value of the E7 was determined to be 0.78 V, which is consistent with results from the literature^47^. Hsu et al. reported similar outcomes of the $V_{th}$ value of the E7 liquid crystal utilizing the T-V graph^19^. Similarly, Nayek and Yi determined that the $V_{th}$ of E7 nematic liquid crystal at 1 kHz frequency was 0.77 V^48^. Furthermore, when the LC molecules’ director is oriented at 45 $^{\circ }$ with regard to the crossed polarizer and analyzer, a bright state is obtained. When LC molecules are rotated by 45 $^{\circ }$ once more, the director aligns parallel to the analyzer, resulting in a dark state^47^.

**Figure 8:**
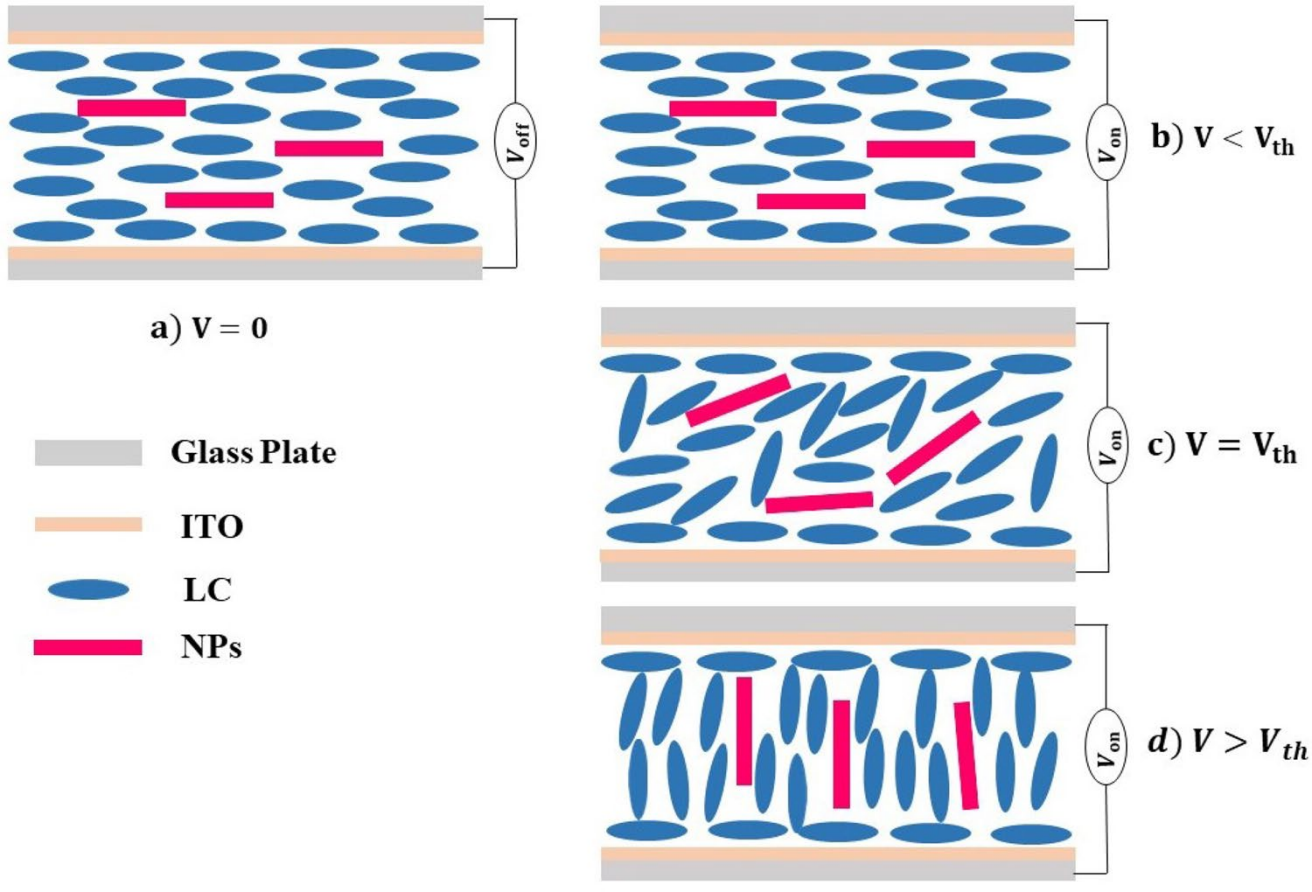
Schematic diagrams for molecules in planar geometry at states without voltage (**a**) $V=0$ and with voltage (**b**) $V<V_{th}$ (**c**) $V=V_{th}$ and (**d**) $V> V_{th}$.

In the beginning, the applied voltage is minimal, and due to anchoring conditions, the LC molecules are aligned in the plane of the LC cell substrate (Fig. 8a,b); this continues until a $V_{th}$ value is reached. When the applied voltage exceeds the $V_{th}$, the orientation of LC molecules changes from planar to homeotropic (Fig. 8c), which is explained by the electrically controlled birefringence effect. If the voltage keeps on rising, the transmittance decreases progressively until it reaches its lowest value. The LC molecules are aligned along the applied electric field due to the reorientation of the molecules (Fig. 8d). It is observed that the minimum and maximum peaks are obtained as the voltage keeps increasing. The reason for these peaks can be explained by the LC cell’s thickness, the sample’s birefringence value, and the wavelength of the laser used in the measurement. As a result, the number of maximum and minimum peaks in the T-V graph varies, which is equal to about $\Delta n.d/ \lambda$. Here, *d* symbolizes the thickness of the cell, $\lambda$ is the wavelength of the light source and $\Delta n$ birefringence^47,49^.The T-V plot for E7 clearly shows two peaks in Fig. 7.

**Figure 9:**
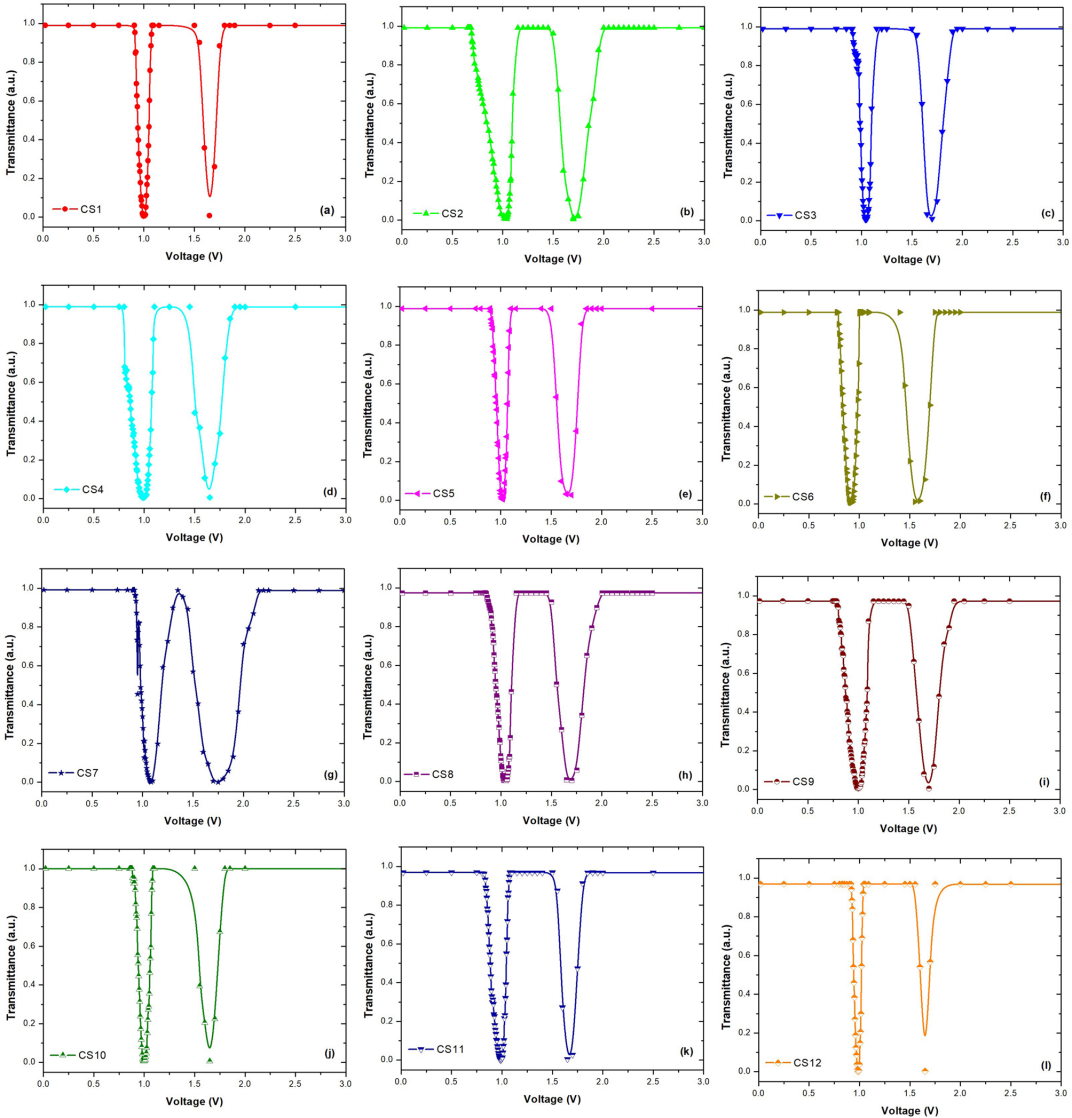
Transmittance-applied voltage plots of composite structures.

The objective of this investigation is to establish composite structures with a lower $V_{th}$ than pure E7. To determine the $V_{th}$ values of 12 composite structures created by different doping ratios of (Al-Cu):ZnO nanoparticles, normalized T-V plots were obtained as in Fig. 9. In a low-voltage range, the transmittance of the composites is nearly constant and the LC molecules are aligned parallel to the LC cell’s substrates and exhibit higher transmittance. However, the transmittance decreases significantly above a certain voltage value as the molecules begin to reorient from planar to homeotropic, resulting in a condition of a dark state or very low transmittance.

**Table 4:** Threshold voltage values of the composites structures.

Composite structures	Threshold voltage (V)
CS1	0.92
CS2	0.71
CS3	0.93
CS4	0.81
CS5	0.92
CS6	0.80
CS7	0.94
CS8	0.90
CS9	0.81
CS10	0.91
CS11	0.86
CS12	0.92

The $V_{th}$ values of the 12 LC composite structures doped with different concentrations of (Al-Cu):ZnO nanoparticle are presented in Table  4. Compared to pure E7 liquid crystal, it was seen that the $V_{th}$ parameter of (Al-Cu)ZnO nanoparticle doped composite structures changed. It is seen that the $V_{th}$ is lower in composite structures where NP2 containing low ratio Al and high ratio Cu are doped in to the E7 at low concentration (CS2 and CS6). Additionally, the $V_{th}$ increases with the increasing concentration of nanoparticles (NP2 and NP4) containing especially high Cu in pure LC. The (Al-Cu):ZnO doped composite system produces an energy barrier as a result of the higher charge density. The doping of nanoparticles into the LC causes to create free electrons, ZnO, Al, and Cu; these free electrons then enter the LC layer, resulting in a rise in charge density along the interface. This situation requires the molecules to have a higher threshold voltage^18^.

### Prediction of threshold voltage using machine learning algorithms

#### Dataset

The experimental dataset consisted of 942 samples (measurements were conducted at an average of 78 different voltage values for each composite structure) with four input features (dispersion rate of Al in ZnO, dispersion rate of Cu in ZnO, dispersion rate of nanostructure in nematic LC and applied voltage value) and one output parameter (transmittance value). Table  5 presents the basic characteristics of the experimental dataset.

**Table 5:** Characteristics of the experimental dataset.

Parameters	Unit	Min value	Max value	Mean
Al rate	%	1	8	4.5
Cu rate	%	1	8	4.5
ZnO rate	%	1	5	3
Voltage value	V	0.01	11.4	2.12
Transmittance value	a.u	0.056	124.4	74.21

#### Fine-tuning

The hyper-parameters of the machine learning algorithms have a significant effect on preventing over-learning and increasing prediction performance. When the hyper-parameters were optimized through the grid search method, for kNN, the best result was attained using the Manhattan distance measure with k = 2. A maximum depth of 13 gave the highest accuracy for DT. For ET, the highest accuracy was obtained when the maximum depth was 20 and the number of estimators was 110. Lastly, the best result for AB was obtained with Extra Trees Regressor estimator using 100 as the base learner.

#### Prediction results

All ML algorithms were performed with the 10-fold cross-validation technique, and the prediction performances of these algorithms for transmittance values were tested using MAE, RMSE, and $R^{2}$.

**Figure 10:**
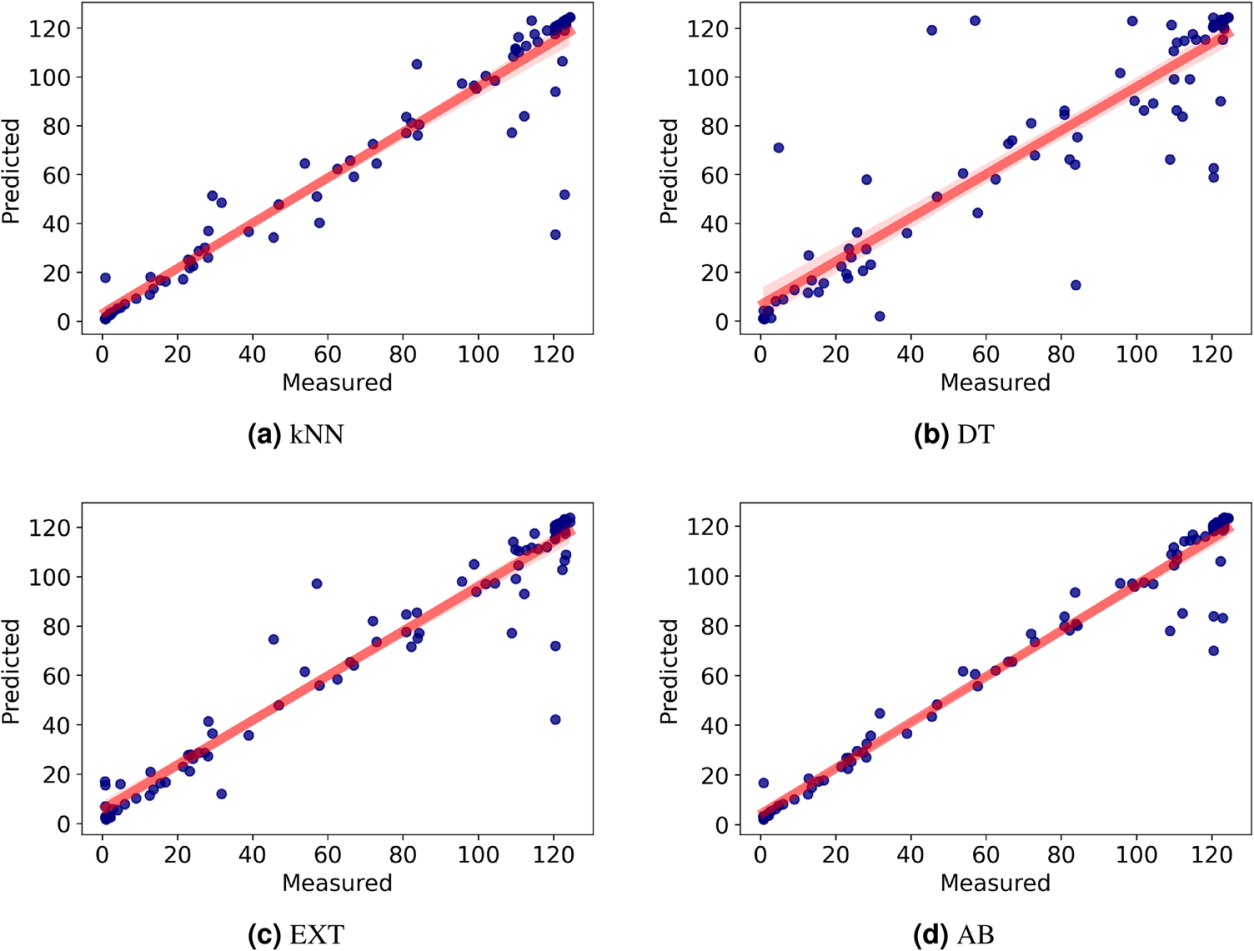
Prediction performance of the algorithms (**a**) kNN, (**b**) DT, (**c**) EXT (**d**) AB.

Figure 10 shows the correlations between the measured and predicted transmittance values. In the graphs, the horizontal axes represent the measured transmittance values whereas the vertical axes represent the values predicted by the algorithms. Based on these results, it seems that the AB algorithm has the highest prediction performance, while DT has the worst prediction performance in predicting the transmittance value.

Table 6 shows the comparison of the MAE, RMSE, and $R^{2}$ values of the algorithms. The table indicates that the $R^{2}$ value of the AB algorithm is over 96. In addition, considering that the average transmittance value in the experimental dataset is 74.21, the MAE value of the AB algorithm (4.44) is at an acceptable level. Thus, it can be concluded that using the AB algorithm, the transmittance values of a composite structure can be predicted with high accuracy.

**Table 6:** Comparison of the algorithms performances for predicting transmittance value.

Algorithms	MAE	RMSE	$R^{2}$
kNN	5.24	13.51	0.92
DT	9.72	19.39	0.83
ET	6.3	12.81	0.93
AB	4.44	9.41	0.96

### On-demand composite structure prediction

The prediction results show that our prediction model using the AB algorithm can predict transmittance values with high accuracy. To determine the concentration ratios of the materials with the minimum $V_{th}$, we performed a brute-force search approach via the developed model. In the brute-force search, the concentration ratios of Al and Cu in (Al-Cu):ZnO change between 1 and 8 (with increment 1), the concentration ratio of (Al-Cu):ZnO in E7 nematic LC changes between 1 and 5 (with increment 1) and the voltage value in electro-optical measurements changes between 0.01 and 10 (with increment 0.005). We predicted the transmittance values for each combination using the AB algorithm trained on the experimental dataset. Then, each composite structure’s $V_{th}$ was estimated as explained in Sect. "Detection of threshold voltage of the composites".

After brute-force search, 26 different composite structures (among 320 different concentration ratio combinations) were found to have lower threshold voltages than the pure nematic liquid crystals. When these composite structures were analyzed, it was seen that the concentration ratio of Al in (Al-Cu):ZnO changed between 1 and 5, and the concentration ratio of Cu in (Al-Cu):ZnO changed between 5 and 8. Furthermore, the concentration ratios of (Al-Cu):ZnO in E7 liquid crystal were 1 and 2. These results are compatible with the literature^18^. Experimentally obtaining all predicted composite structures is not practically meaningful in terms of cost and time. Hence, we considered the composite structures with concentration ratios of 1, 5 and 8 for Al and Cu in (Al-Cu):ZnO and 1 and 2 (Al-Cu):ZnO in E7 liquid crystal since these were the limits and most common values in predictions. Therefore, we decided to create 3 new structures that meet the prescribed conditions.

Table  7 lists the related composite structures. These three new composite structures proposed by the ML predictions were experimentally produced using the same procedures in Sects. "Synthesis of co-doped ZnO Nanoparticles" and "Preparations of the nanoparticle doped LC composites". For this purpose, new nanoparticles for NP5 ((1% Al–5% Cu):ZnO) and NP6 ((5% Al–8% Cu):ZnO) were synthesized using identical experimental procedures. Then, new composite structures CS13, CS14 and CS15 were obtained by doping 1% NP5, 1% NP6 and 2% NP2, respectively. Finally, the electro-optical performance of liquid crystals was assessed by utilizing an electro-optical transmittance system.

**Table 7:** Predicted and measured threshold voltage values of the ultimate composite structures.

Number	Composite structure	Predicted $V_{th}$ (V)	Measured $V_{th}$ (V)
CS13	E7+1%NP5	0.71	0.63
CS14	E7+1%NP6	0.72	0.84
CS15	E7+2%NP2	0.72	0.77

Table  7 also presents the predicted and measured $V_{th}$ values. It can be observed that 2 of 3 materials suggested by machine learning (CS13 and CS15) have a lower $V_{th}$ than the pure LC (0.78). Furthermore, CS13 has the lowest $V_{th}$ voltage among all manufactured composites, with a $V_{th}$ value of 0.63. With a 1%NP5 doping concentration, the greatest decrease in $V_{th}$ is 19%.

**Figure 11:**
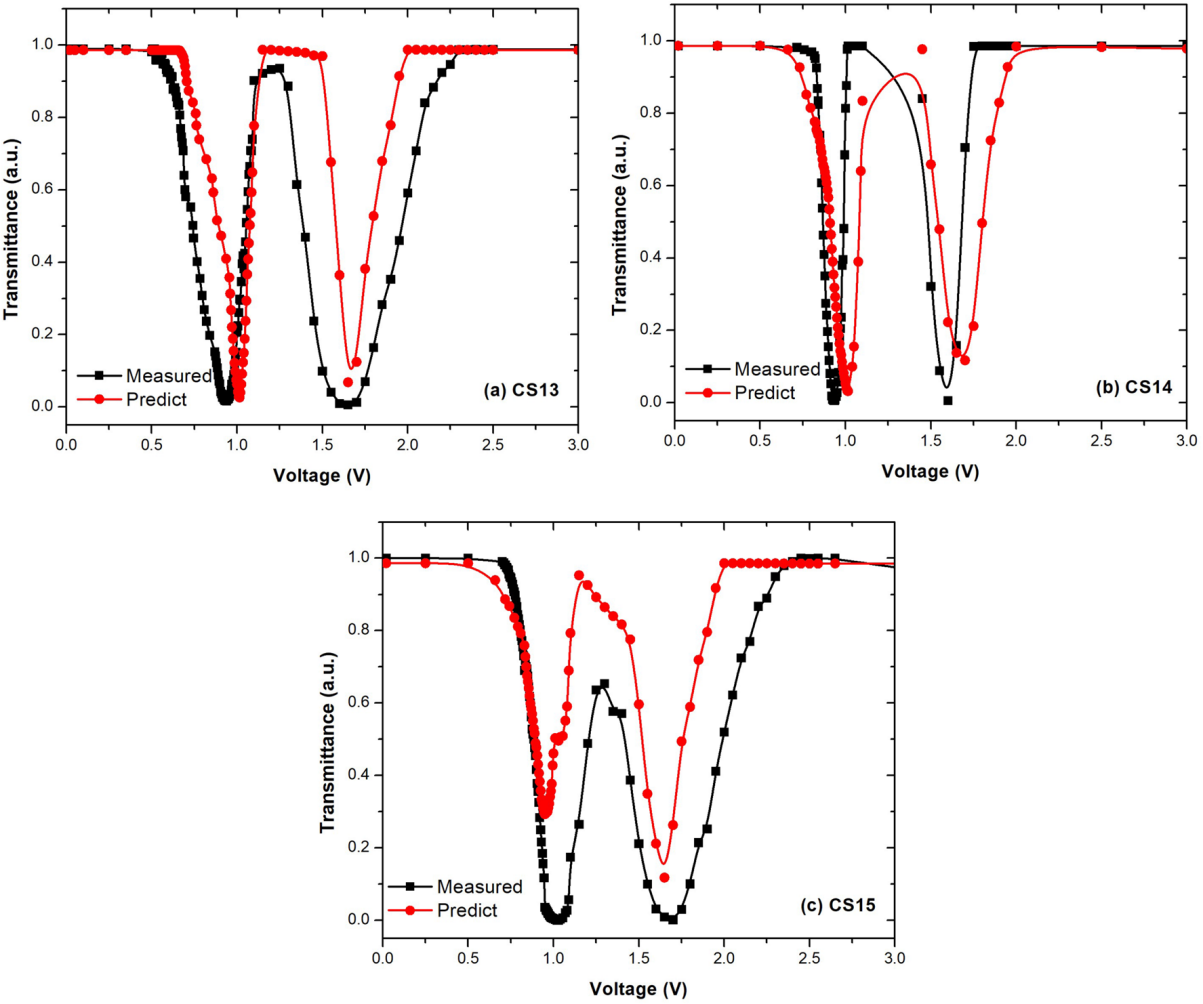
Comparison of predictions with the experimental data for T-V plots of the ultimate composite structures.

Figure 11 presents the measured and predicted transmittance values of CS13, CS14 and CS15. This figure demonstrates that the predicted transmittance values are highly consistent with experimental data for all three composite structures. Furthermore, it is seen that the developed prediction model can detect the first sharp decrease in transmittance values of the composite structures with high accuracy.

## Conclusion

Producing materials with desired properties is a fundamental task in material science. In this work, we aimed to minimize the $V_{th}$ of the co-doped ZnO doped LCs. The biggest challenge in this direction is determining the ideal concentration ratios of the materials used to form the composite structure. We used different machine learning algorithms to determine the ideal concentration ratios of the materials for the minimum $V_{th}$. In this manner, four NPs containing different ratios of Al and Cu were synthesized, and twelve new composites were produced by doping the pure E7 with different concentration ratios of these NPs. Voltage-dependent transmittance data of the 12 composite structures were obtained by Utilizing the electro-optical transmittance system. Using pre-determined concentration ratios, the $V_{th}$ of pure LC was reduced by 9% (CS2). Then, we developed a prediction model for estimating the transmittance values of these composite structures. By training the prediction model on the experimental dataset, we estimated the transmittance values of composites with different concentrations that were not produced experimentally and calculated the $V_{th}$ value of these composites. After determining the ideal concentration ratios for the composites with the minimum $V_{th}$, three new composite structures (CS13, CS14, and CS15) were produced. Among these structures, the best result was obtained with CS13. The $V_{th}$ value, which was 0.78 V for pure LC, was reduced to 0.63 by adding 1% NP5 to the LC. Using the concentration ratios determined by the machine learning algorithm, the $V_{th}$ of pure E7 liquid crystal was reduced by 19% (CS13).

In this work, we have shown how to combine experiments with machine learning-based prediction algorithms to determine specific materials with certain desired properties. This work also demonstrates how carefully created materials data can be used to train machine learning models. The model used in this study merely requires the concentration ratios of the materials to estimate the properties of the composite structures. The material design methodology applied in this work can be used for any class of composites as long as there is sufficient data available for training. Lastly, it should be noted that all property predictions from the on-demand prediction model come with some uncertainties, which are unavoidable in any learning method. Nevertheless, we have developed a promising composites design methodology that can actively pursue specific composites that would suit desired requirements.

## Data Availability

The datasets used and/or analyzed during the current study are available from the corresponding author upon reasonable request.
